# A population based study of hospitalised seriously injured in a region of Northern Italy

**DOI:** 10.1186/1749-7922-8-32

**Published:** 2013-08-12

**Authors:** Osvaldo Chiara, Cristina Mazzali, Sofia Lelli, Anna Mariani, Stefania Cimbanassi

**Affiliations:** 1 Trauma Team Dip. DEA-EAS, Ospedale Niguarda Ca’Granda, Piazza Ospedale Maggiore 3, Milan, 20162, Italy; 2 Quality Department, Ospedale Niguarda Ca’Granda Milan, Milan, Italy; 3 Universita’ di Milano, Dip, Scienze cliniche Luigi Sacco, Milan, Italy

**Keywords:** Epidemiology, Major trauma, Population-based study, Trauma registry, Trauma system

## Abstract

**Background:**

Injury is a public health problem in terms of mortality, morbidity and disability. The implementation of a regionalised trauma system has been proved to significantly reduce the social impact of severe trauma on population. A population-based registry may be useful to obtain reliable epidemiologic data.

**Aim:**

To perform an exhaustive analysis of severe trauma patients hospitalised in Lombardia, a region of northern Italy.

**Materials and methods:**

The regional Hospital Discharge Registry (HDR) was used to retrieve data of all patients who suffered from serious injuries from 2008 to 2010. ICD9-CM codes of discharge diagnoses were analysed and patients coded from 800.0 to 939.9 or from 950.0 to 959.9 have been retrieved. Femur fractures in elderly and patients with length of hospital stay less than 2 days were excluded. Patients have been considered seriously injured if discharged dead or any of followings: admission or transit in ICU, need of mechanical ventilation, tracheotomy, invasive hemodynamic monitoring. Average reimbursement based on DRG has been evaluated.

**Statistics:**

Student’s *t* test, ANOVA for continuous data, chi-square test for categorical data were used, and a p value less than 0.05 was considered significant.

**Results:**

The severely injured patients hospitalised in Lombardia in three years were 11704, 391 per million per year. Overall mortality was 24.17% and increased with age. Males aging from 18 to 64 years had more occupational injuries, trauma on the road and violence by others. Females were more susceptible to domestic injuries and self inflicted violence, mostly in older ages. Acute mortality was higher after traffic accidents, while late mortality was increased in domestic trauma. Pediatric cases were unusual. A significant increase (+10.18%) in domestic trauma, with a concomitant decrease (-17.76%) in road-related accidents was observed in the three years study period. Reimbursement paid to hospitals for seriously injured was insufficient with regard to estimated costs of care.

**Conclusion:**

Serious injury requiring hospitalisation in Lombardia is still an healthcare problem, with a trend toward a decrease of traffic accidents, increase in domestic trauma and involvement of older people. These results may help to plan a new regionalised Trauma System.

## Introduction

Injury is a major public health problem in terms of mortality, morbidity and disability and it has been largely demonstrated that the organisation of a regionalised Trauma System significantly decreases the deleterious impact of severe trauma on population [1,2]. In Europe the inclusive trauma system model has gained dominance. In this model a network of hospitals with different resources takes care of trauma patients suffering from any among the full spectrum of injuries [3]. Epidemiologic information based on the entire population in a given region and understanding the number of severely injured that need to be admitted to a level one hospital, is of pivotal importance in the design of an inclusive Trauma System. With this objective, methodological approaches in measuring incident rates should use large representative samples of the whole population, to offer the potential to observe data on all the people living in a region or a nation. Trauma registries contain detailed information, but this is offset by the limitation of including only patients treated at trauma centre and already triaged as “severe” at a dedicated trauma unit. On the contrary, population-based registries have usually been recorded for many years and are available for time periods before changes of the Healthcare system. Additionally, they contain readily available, alphanumeric-coded information and allow easy and low cost analysis. Moreover, population-based registries may be used to investigate resources consumption and evaluate costs of the system. Recently, many investigators have started to use large databases for quality assessment studies in trauma care, and these works are classified as providing “high end” Class III evidence [4-8].

The objective of this study was to perform an exhaustive analysis of severe trauma patients hospitalised in Lombardia, a mixed rural/industrial region of northern Italy. The hospital discharge registry, a population-based record of all hospitalised people of the country, has been used as source of data. All hospital admissions for injuries during a three years period have been included and severely injured patients have been extrapolated. This analysis may be a useful starting point for evaluating the need for resources and costs of regional Trauma System implementation.

## Methods

Lombardia is a mixed rural/industrial region of the northern Italy, with an area of 24,000 Km [2] (9,302 square miles), with Alpi Mountains in the north and hill or flat in the south. Residents, evaluated at the end of 2010, were 9,737,074 (1,046 persons per square mile), 48.87% males, and Milano is the capital city. In this Region there are nine hospitals which function as level 1 or 2 Trauma Centre. The regional Hospital Discharge Registry (HDR), a part of the national HDR, includes the discharge forms of all hospitalised patients of the region since 2001. A common minimum data set, including demographics, place of residency, hospital length of stay (LOS), wards of admission or transit, discharge diagnoses, therapeutic procedures, and outcome, is adopted for all of the public or private hospitals partially or totally financed by the Regional Health Service (97% of existing hospitals). In HDR discharge diagnoses (one principal and up to five secondary diagnoses) and procedures are coded using the Clinical Modification of the International Classification of Diseases 9th edition (ICD-9-CM). In-hospital deaths are all recorded in HDR.

Reimbursement of public or private hospitals is calculated by Government of the Region using the disease-related group (DRG) system and the discharge form of HDR is the administrative document used to calculate the DRG: each patient is weighted on the sequence of ICD-9-CM diagnoses, therapeutic procedures, complications and associated morbidities and the value of assigned DRG is reimbursed to the hospital.

### Data extraction

To conduct this study all hospital admissions in Lombardia during a period of three years, from 2008 to 2010, have been reviewed. The aim was to select from regional HDR all patients who suffered from serious injuries.

All patients with at least one principal or secondary diagnosis coded from 800.0 to 939.9 or from 950.0 to 959.9 have been considered. Burns, scalds and frostbites, chemical corrosion, poisoning, intoxication, drowning and hangman, suffocation, electrocution, radiation and medical treatment complications, have been excluded. Furthermore, femur fractures (820.0 and 821.9), as the only traumatic diagnosis, have been considered only if affecting people younger than 65, to exclude femur fractures of elderly due to osteoporotic complications. All patients have been coded with an individual number. Patients with the first admission in a rehabilitation or spinal unit, with a LOS less than two days, unless discharged dead or transferred from or to other facilities, have been excluded.

To select seriously injured any of the following criteria have been used:

• patients discharged dead

• patients admitted in intensive care unit (ICU) during the course of hospital stay

• patients which have been mechanically ventilated (ICD9 code 96.70-96.72) or received tracheotomy (31.1-31.29)

• patients which received invasive hemodynamic monitoring (89.60-89.69)

All patients with at least one of these characteristics have been classified as serious trauma and included in the analysis.

Distribution of severe trauma for specific age-sex population groups has been estimated. The modality of trauma has been identified as:

• accident at workplace

• accident in domestic pertinence

• road-related accident

• assault (violence inflicted by others)

• self-inflicted violence

• other

The Regional Health System allowed the analysis of the reimbursements, giving the investigators the value in euros of DRG assigned for each case. In order to characterise the time distribution of trauma deaths, from HDR it was possible to classify hospital deaths into acute (within two days following admission), early (from three to seven days), or late deaths (more than seven days).

### Statistics

Data processing and statistical analysis were performed using SAS 9.2^®^. Continuous data were compared by ANOVA or Student’s *t*-test, while categorical data were analysed using chi-square test. Differences for all tests were considered significant with a p value less than 0.05.

Incidence rates for severe trauma hospital admission in specific age-sex population groups were calculated, using the resident population estimated at the end of administrative year 2010.

## Results

**Figure 1:**
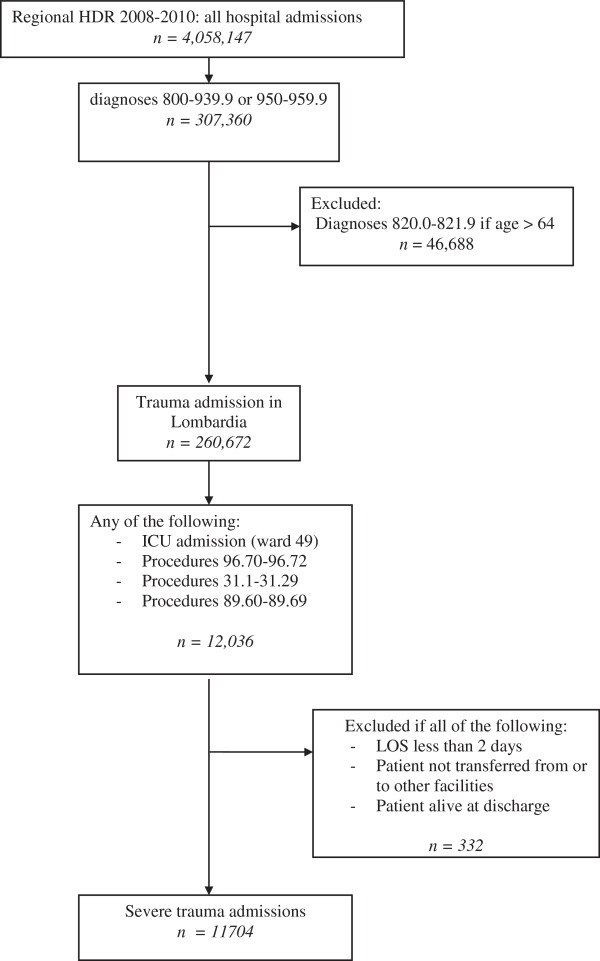
Algorythm for the inclusion in the epidemiologic analysis.

Selected records have been 12,036 from which 332 cases (2.76%) have been excluded because LOS <2 days, not deceased or transferred (Figure 1). Finally, the working database of in-hospital severe trauma counted 11,704 cases, 892 (7.62%) non-residents in Lombardia.

Table 1 describes general results of data extraction. The severely injured patients hospitalised in Lombardia during a three years period were the 0.80% of all hospital admissions, on average, 391 cases per million inhabitants per year. Males constituted 65.13% of these cases and showed a significantly longer hospital LOS, ICU-LOS and rate of admission in ICU. Males showed also a higher value of reimbursement. Overall mortality was 24.17%, with an incidence rate of 9.68/100,000 per year. Surprisingly, mortality of males was lower than in females. Significant differences of these variables during the three years of the survey were not appreciated. The calculated incidence rate of hospital admissions for severe trauma was 40.06/100,000 inhabitants per year (27.33 for females and 53.38 for males). The highest rate was observed in the over 74 years age group (129.21/100,000), while the lowest for children between 7 and 12 years (11.73/100,000).

**Table 1:** Severe trauma patients hospitalized in Lombardia

	***Number***	***Deceased***	***% deceased***	***Hosp. LOS (±SD)***	***% ICU adm***	***ICU LOS (±SD)***	***Avg remb (€) (±SD)***
***Total***	***11704***	***2829***	***24.17***	***18.53 (18.89)***	***74.09***	***6.12 (11)***	***13˙759.82 (19347.55)***
**Male**	7623	1588	20.83*	19.35* (20.43)	80.44*	7.02* (11.71)	15˙128.02* (20464.93)
**Female**	4081	1241	30.41	17.00 (18.73)	62.22	4.43 (9.32)	11˙204.13 (16771.51)
**Year 2008**	3866	954	24.68	18.77 (20.31)	74.70	6.21 (11.40)	13˙684.64 (18821.89)
**Year 2009**	3960	961	24.27	18.48 (19.65)	73.79	6.25 (11.36)	13˙757.31 (19199.84)
**Year 2010**	3878	914	23.57	18.34 (19.70)	73.78	5.90 (10.20)	13˙837.34 (20008.15)

LOS: length of hospital stay. Avg remb: average rembursement in euros based on disease related group (DRG). ICU adm: admission in intensive care unit. SD: standard deviation. * p < .001 males vs females.

In Table 2 patients have been analysed according to age groups. Overall, severe trauma affected adults: 4206 cases in age 0–45, 7495 cases after 45 years. Mortality increased with age, reaching nearly 50% in trauma victims older than 75 years. Similarly, hospital and ICU-LOS, rate of admission to ICU and reimbursed DRG, all increased with age, with the higher levels in ages between 13 and 74 years. On the contrary, pediatric cases (age group 0–12) were only 482 in three years, with shorter ICU LOS, decreased mortality and lower levels of reimbursement. All of these differences were statistically significant (p < .0001, ANOVA).

**Table 2:** Severe trauma hospitalized in Lombardia according age groups

			***Modality of trauma: absolute values***				
***Age groups***	***Number***	***Deceased***	***%_ deceased***	***LOS (±SD)***	***% ICU adm***	***ICU LOS (±SD)***	***Avg remb (€) (±SD)***
**00-06**	322	15	4.65	10.65 (15.22)	79.165	3.36 (7.49)	6˙588.98 (11828.14)
**07-12**	160	4	2.50	12.50 (12.74)	88.75	3.88 (7.81)	7˙492.89 (10229.22)
**13-17**	411	19	4.62	17.20 (15.94)	95.38	6.39 (9.20)	12˙908.43 (16509.47)
**18-45**	3313	334	10.08	20.88 (21.35)	93.96	7.66 (11.25)	16˙144.73 (19550.47)
**46-64**	2148	356	16.57	21.01 (22.31)	85.52	7.57 (12.74)	16˙207.54 (21784.13)
**65-74**	1657	407	24.56	20.39 (21.06)	74.83	7.13 (11.93)	16˙224.24 (21679.17)
**>74**	3690	1693	45.88	15.21 (16.34)	45.85	3.74 (9.20)	10˙067.29 (16701.65)

All differences significant (p < .0001) at ANOVA.

In three cases age informations have been missed.

The cause of accident has been indicated in 72.98% of cases (Tables 3 and 4) and “other mechanism”, road-related trauma, injured in domestic pertinences and at workplace were the principal conditions. As expected, accidents on the road and at the workplace were the principal causes of trauma for males aging from 18 to 64 years. On the contrary, accidents in domestic pertinences increased with age, being the principal cause of trauma after 64 years, and old women were affected the most. Violence inflicted by others (assault) or self-inflicted violence was rare in Lombardia and affected people 18 to 64 years old. In pediatric age most of cases were domestic or road-related. Statistic analysis demonstrated a significant association at chi-square test between gender and modality of trauma: males had more occupational injuries, trauma on the road and injuries caused by violence by others, while females were more subjected to domestic injuries and self inflicted violence.

**Table 3:** Distribution of patients according to age groups, gender and modality of trauma

			***Modality of trauma: absolute values***					
***Age groups***	***Gender***	***Missing***	***Work***	***Domestic***	***Road***	***Assault***	***Self inflicted violence***	***Other mechanisms***
**00-06**	**m**	32		48	44	1		82
	**f**	30		24	14			47
**07-12**	**m**	3	1	3	49			53
	**f**	3		1	21	2		24
**13-17**	**m**	16	2	7	200	2	2	92
	**f**	9		3	48		8	22
**18-45**	**m**	173	301	49	1479	122	70	525
	**f**	75	13	15	307	18	35	131
**46-64**	**m**	255	202	93	518	20	28	456
	**f**	137	2	67	145	11	31	183
**65-74**	**m**	311	21	141	174	5	13	392
	**f**	191	1	96	113	1	8	190
**75-**	**m**	538	3	289	193	3	7	602
	**f**	717	2	494	122	3	4	713
**Total(1)**		**2490**	**548**	**1330**	**3427**	**190**	**207**	**3512**

**Table 4:** Differences between male and female for modalities of trauma were significant at chi square (p < .0001)

***Chi square***	***Work***	***Domestic***	***Road***	***Assault***	***Self inflicted***	***Other***	***Total***
***Male***	530	630	2657	155	121	2202	**6295**
***Female***	18	700	770	35	86	1310	**2919**
***Total***	**548**	**1330**	**3427**	**190**	**207**	**3512**	**9214**

(1) In three patients (2 assault and 1 self inflicted violence) age was not available.

Furthermore, the age of exposure to injuries changed with gender. The mean age of females involved in domestic, road-related trauma and in the category of other modalities was significantly higher (Table 5). Age between gender was not different in accidents during working activities and injuries derived from violence. Same differences of age between gender were evident also in deceased patients (Table 6). Women who died after trauma were significantly older when the cause of death was an accident at work, on the road, violence by others or self-inflicted, other mechanisms.

**Table 5:** Differences between age, gender and cause of trauma (SD, standard deviation)

	***Male***			***Female***		
***Trauma modality***	***#***	***Mean age***	***SD***	***#***	***Mean age***	***SD***
**Work**	530	42.51	13.00	18	41	21.09
**Domestic**	630	65.30	24.17	700	75.67*	18.95
**Road**	2657	39.31	19.63	770	46.51*	23.60
**Assault**	155	35.61	14.27	35	41.49	18.67
**Self inflicted violence**	121	44.61	17.89	86	45.01	16.41
**Other**	2202	55.12	24.65	1310	67.43*	23.86

* p < .0001.

**Table 6:** Age of deceased patients according to cause of trauma and gender

	***Male***		***Female***	
***Cause of trauma***	***#***	***Mean ± SD***	***#***	***Mean ± SD***
**Missing**	405	72.66 16.72	383	79.83 13.28
**Work**	44	43.14 14.10	2	61.5* 40.31
**Domestic**	223	76.86 14.99	268	82.15 11.69
**Road**	355	50.58 22.57	140	60.53* 21.51
**Assault**	23	43.57 17.46	5	60.00* 14.63
**Self inflicted**	29	49.43 22.30	15	53.20* 14.34
**Others**	509	71.92 17.46	428	80.49* 12.28
**Total**	1588	71.48 17.80	1241	77.95* 15.57

* = p < .001.

Time distribution of deaths changed with cause of trauma (Table 7). Late deaths were more often represented in domestic trauma and in the category other mechanisms. On the contrary, deaths at work, on the road and after violence were acute in the majority of cases. Females and older age people showed a tendency to increase in late deaths, although not significantly. In late deaths of patients older than 64 years a systemic complication was the principal diagnosis in 51.4% (pulmonary or cardiovascular failure, mainly), while it was only 17.6% in victims younger than 64. The overall rate of patients admission to one of the nine level 1 or 2 hospitals was 41.58%, but this percentage decreased to 29% in patients older than 64. The mortality was 17.75% in level one or two hospitals, while it was increased to 27.95% in local – non trauma center hospitals.

**Table 7:** Time distribution of deaths in deceased patients

	***Total #***	***%***	***Age (±SD)***	***% male***	***Work %***	***Domestic %***	***Road %***	***Assault %***	***Self inflict %***	***Other %***
***Acute***	1111	39.27	64.13 (23.19)	60.21	63.04	35.44	67.47	64.29	75.00	33.40
***Early***	658	23.26	77.00 (16.00)	52.12	17.39	27.70	13.74	10.71	9.09	27.85
***Late***	1060	37.47	75.76 (15.17)	54.33	19.57	36.86	18.79	25.00	15.91	38.74

**Figure 2:**
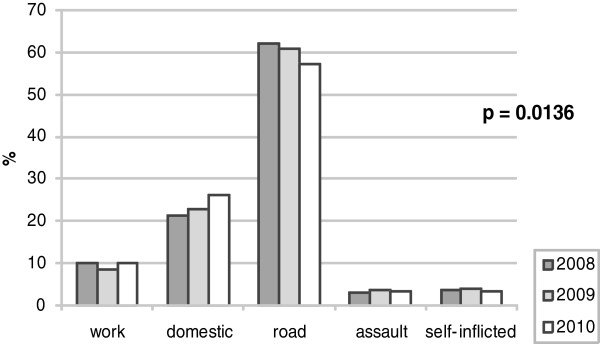
Trends of causes of trauma during the three years of the study.

Figure 2 shows trends of causes of trauma during the three years of the survey. A significant increase in domestic trauma (from 422 in 2008 to 465 in 2010, +10.18%), with a concomitant decrease in road-related crashes (from 1233 to 1014, -17.76%) were observed.

## Discussion

### Methods of selection

The aim of this study was to perform an exhaustive analysis encompassing the whole population in Lombardia and to identify the number of seriously injured people who need hospital admission. It is the first time in Italy that a population-based registry has been used to investigate hospitalisation of major trauma in order to design a regionalised Trauma System. A previous study [8] in our country used national HDR to investigate epidemiology of trauma deaths.

A non-integrated Trauma System, such as in Lombardia, implies that many trauma patients are treated in non-trauma hospitals and the use of specialised trauma registries for epidemiologic studies in these conditions excludes patients who receive definitive treatment in non-Trauma Centre hospitals. In our survey less than fifty percent of cases were admitted in one of the nine hospitals which function as level one or level two Trauma Centres and this observation confirms the choice of an administrative database to obtain population-based data.

The methodological approach of cases selection in the present study may be debated. Hospital databases contain ICD diagnoses which lack information about injury severity. On the other hand, specialised trauma registries, in line with international conventions, use the Abbreviated Injury Scale (AIS), an anatomically-based injury description system which allows computation of ISS, or New Injury Severity Score (NISS) the most reliable and extensively used measure of injury severity [9]. In the middle of 1990s Osler et al. introduced the ICD9 based ISS (ICISS) that allows severity to be classified based on the ICD9 classification of injuries [10]. There is limited evidence of the validation and performance of ICISS in epidemiologic studies [11,12]. ICISS is a product of survival risk ratio from each injury sustained, based on the values of the survival rates of prior patients with similar diagnoses as classified by ICD9. Validity of ICISS derives from accuracy in compilation of list of diagnoses. In Italy hospital discharge forms mainly fulfil an administrative purpose and the sequence and choice of listed diagnoses may be determined in combination in order to generate the DRG that provides maximal payment. As a result of these limitations we considered inappropriate a retrospective analysis of regional HDR for an epidemiologic study on serious injury. We preferred to consider all hospitalised trauma patients of Lombardia with an “ex-post” selection of severity based on procedures unequivocally used in critically injured (ICU admission, mechanical ventilation, tracheotomy, invasive monitoring), or based on the fatal outcome during hospital stay. Unfortunately, vital signs, number of transfusions, laboratory values were not available in HDR. A possible selection bias is the inclusion of patients with minor trauma and severity due to complications or associated illnesses. However our focus was the use of hospital resources and a patient with minor trauma and concomitant severe illness needs in any case to be triaged to a level one Trauma Centre.

### Epidemiology of serious injury

Severe trauma patients hospitalised in Lombardia have been on average 391 per million inhabitants: because in the trauma deaths study [8] we observed a proportion of out-of-hospital deaths (on site and in emergency department) of 38% in the capital Milano during 2007. This suggest that in the regional area the Emergency System, pre-hospital and in-hospital, has to manage about 5258 major trauma patients per year, 540 per million inhabitants. This datum may be overestimated because it considers as the denominator only the resident population and the 7.62% of seriously injured patients at the numerator were non-residents in Lombardia. However, it is not possible to calculate transients or tourists of the Region. The resulting number of 540 major trauma patients per million is analogous to that described by Di Bartolomeo et al. in a study, based on specialised trauma registry, in a north-east region of Italy [13] with 1,200,000 inhabitants, an established Trauma System and only two Trauma Centres receiving major trauma. The Italian data of both these studies are higher than those showed in other European countries, as Mersey-Wales [14] and Ireland [15] but lower than United States reports [16,17]. The selection criteria used in this study seem to be appropriate: all trauma patients who needed ICU treatment or who died during hospital stay have been included. A possible explanation of differences between Italian and US data may be the lower rate in Europe of interpersonal violence. Severe trauma admissions in Italy are due to blunt trauma in 94% (in Lombardia more than 97%), with less than 17% of surgical cases for torso injuries [18]. These observations outline the need of a reduced number of Trauma Centres, to obtain local concentration of cases and surgical skill.

The hospital mortality in Lombardia of 24.17% (incidence rate of 9.68/100,000) is lower than that described in overall Italy in 2002 in the national trauma death study [8] (14.5/100,000) and comparable with the data recorded by Creamer et al. in Auckland in 2004 [19].

Analysis according age groups demonstrates that the highest number of severe trauma occurs in old adults, while pediatric cases are unusual. An increasing average of the age of the victims of serious trauma is common in Western countries studies [20].

The high mortality of our study needs to be discussed. Less than half of trauma patients have been admitted to level one or two hospitals and this percentage was further reduced in patients older than 64. This is a common result in many epidemiologic studies. Ciesla et al. [21] observed that access to a designated trauma centre was dependent on proximity for severely injured elderly, while distance from trauma centre did not limit admissions for children and adults. Hsia et al. [22] demonstrated that the odds of admission to a trauma centre decreased with increasing age.

In Lombardia the percentage of hospital deaths has been higher in non level one or two hospitals: the lack of local expertise, reduced technology as well as unavailability of specialists are recognized causes of increased trauma mortality. At the time of the study a regionalized trauma system did not exist, triage protocols for centralization of severely injured were not uniformly applied and a formal hospital trauma team organization was active only in one hospital of the region. Moreover, severely injured older than 64 were the 46% of study population, with the highest hospital death rate (from 25% to 46%). All these considerations may explain why the mortality presented in this Italian study is higher than other reports [23]. During the late 2012 a new law has formally instituted in Lombardia the regional trauma system. Now, efforts are needed to determine trauma resources and triage protocols and this study may be helpful to this project.

A special consideration is due to the severe trauma in the elderly, in terms of amount of resources expended with regard to the level of functional recovery. Recently, Grossman et al. [24] demonstrated an appreciable acute survival (66% or 69%, with or without brain injury) for geriatric trauma patients (>64) admitted to a level one trauma centre with an ISS > 29. Moreover, a good long term recovery has been observed in 67%. The prolonged life expectancy and active life style of many elderly, the increasing number of severe trauma after 64 years, together with promising results of modern trauma care, suggest the use of significant resources also in geriatric trauma, although with specific protocols to avoid futility.

### Causes of trauma

Evaluating the causes of trauma, a precise definition in our study has been possible only in half of cases: in 21.27% the datum has been missed (i.e. not indicated on hospital report) while in 30% the category “other mechanism” has been assigned. Nevertheless, it is possible to make some observation in more than five thousands of cases for whom cause of trauma was precise and available. Young-adult males have been more exposed to road related accidents, while females in old age have been principally victims of unintentional domestic injuries. These results are consistent with other epidemiologic surveys [25-27]. Moreover, the age of injured females has been higher for all causes of injury and the same has been also observed in fatal trauma. Because trauma mortality increased with age, this might explain why in Lombardia trauma mortality in women has been globally higher than in men.

Another important observation is the trend of causes of trauma during the three years of the study. The 17.76% decrease in road-related injuries demonstrates that primary and secondary prevention programs for car, motorcycle, pedestrian, cycling accidents have obtained appreciable results. On the contrary many efforts need to be made for trauma prevention in houses, particularly of falls in old women living at home. The design of a new Trauma System must take into account these data: the new challenge will be the need to treat an increasing number of serious injuries in elderly people, with all the problems of concomitant illnesses, complications, prolonged ICU and hospital LOS, increased costs of healthcare and need of complex rehabilitation programs for the social reinstatement. On the other hand, pediatric cases are less than 200 per year in ten millions inhabitants and injured children need to be centralized in few highly specialised centres.

The low number of trauma due to violence underlines a significant difference in trauma epidemiology between Europe and overseas Countries. In Lombardia only 2.06% of serious trauma (where the cause has been formally indicated) were consequence of assaults (both penetrating or blunt) and this amount is sharply lower than North America [28]. However, media reports of stabbing and shootings and anecdotal evidence based on presentations to the emergency departments support the idea that interpersonal violence is on the rise, particularly between immigrates from Asia and Africa, as also observed in other countries [29].

Finally time distribution of hospital trauma deaths demonstrated that acute and early deaths regarded principally road-related injuries, trauma at workplace and assaults or self-inflicted violence. On the contrary, late deaths increased in victims of domestic trauma. Differences in age between victims with acute-early deaths and victims of late deaths suggest that young patients demise has been related in the acute – early phase to the severity of injuries, while elderly people died principally for related complications [30]. These observations are consistent with the results obtained also in the national trauma deaths study [8].

A late mortality close to 40%, mostly related to domestic trauma in elderly, is a substantial change and may impact significantly costs of trauma care. Notwithstanding the highest mortality, a reduced rate of ICU admission has been observed in patients older than 74. Although the datum was not available, this may suggest use of resources weighted on functional recovery possibilities. Again, this observation outlines the need of further studies to define protocols of care in this category of patients.

### Funding of trauma system

In Italy, as in many other Countries, public or private hospitals are reimbursed using the DRG system. Data of Lombardia showed that the average amount paid to hospitals for seriously injured patients has been € 13,759.82 per patient. Trauma Systems in Europe demonstrate a significant country-by-country variation of costs, which is in part explained by the level of economic resources available for trauma care [31]. Iapichino et al. demonstrated [32] in a prospective Italian cohort study that variable costs of ICU for poly-trauma amounted € 4,423 per patient. In the UK [33], Sikand et al. examined hospital costs in poly-trauma patients, indicating a cost for the initial hospital LOS of € 20,408 per patient. Morris et al. [34]. In an international clinical trial about blunt trauma reported an average cost of 37,914 for initial hospital care. In general, ICU stay accounted for the majority of costs and other significant resource use included transfusion requirements and surgical procedures. Moreover, fixed costs of emergency care hospitals, rescue management and rehabilitation of trauma victims consume healthcare resources considerably. These data suggest that average reimbursement based on DRG for serious injuries which has been paid in Lombardia has been largely insufficient. Determining the cost-effectiveness of trauma interventions requires accurate data on the fixed and variable costs and outcome for trauma victims. This process is fundamental in the design of regionalized Trauma System where major trauma patients are concentrated in few specialised hospitals capable of high quality definitive care which need to be adequately budgeted for trauma capacity.

### Strengths and limitations

The strength of this study was the use of a sample that is representative of all claims for a serious injury in a given Region, obtained from a population-based source at the individual level, coupled with demographics and causes of injuries. These data were used to analyse the incidence rates, mortality, type of accidents across different age groups, for men and women, with different patterns emerging for various population groups. The weakness of the study may be the quality of the sanitary data, with the limit that serious injuries number may be only indirectly derived and not calculated from a specific anatomic score. However, the incidence rates of serious injury which have been derived in this study are comparable with those calculated in another Italian study using trauma registry and this represents a confirmation of the reliability of data extraction.

## Conclusions

This study, although with an indirect evaluation of patient severity, has demonstrated that seriously injured who need hospital admission in Lombardia still represent a consistent healthcare problem. Road-related injuries in young-adult males are the principal causes of severe trauma, with a significant acute and early mortality, but there is a tendency toward the increase of elderly people, particularly females, who are exposed to serious domestic trauma, characterised by an elevated late mortality.

These results may be useful for the planning of a new regionalised Trauma System, adequately designed on epidemiologic evidences.

## Competing interests

The authors declared that they have no competing interests.

## Authors’ contributions

OC and SC carried out the study design, CM and SL performed statistical analysis, AM retrived the data. All authors read and approved the final manuscript.
